# Data on microbiological quality of raw cow milk in East Azerbaijan province, Iran

**DOI:** 10.1016/j.dib.2018.10.161

**Published:** 2018-11-03

**Authors:** Payam Safaei, Fatemeh Seilani, Seied Reza Sajedi, Mohadeseh Pirhadi, Afsaneh Mohajer

**Affiliations:** a Department of Environmental Health Engineering, School of Public Health, Tehran University of Medical Sciences, Tehran, Iran; b Student׳s Scientific Research Center, Tehran University of Medical Sciences, Tehran, Iran; c Department of Food Science and Technology, Faculty of Agriculture, Tabriz University, Tabriz, Iran

**Keywords:** Raw milk, Total bacterial count, Somatic cell count, East Azerbaijan province

## Abstract

Microbial contamination of milk can lead to undesirable effects on texture, color, odor, or flavor that result in shorter shelf life. It may also cause serious illnesses in consumers if it contains over than standard limit of these parameters. In this data, we evaluate the total bacterial count (TBC) and somatic cell count (SCC) of raw milk in East Azerbaijan province using BactoScan and Fossomatic equipment, respectively. According to the 30 points selected in the province map, the 10,800 samples were collected during a one-year period. Microbiological results in this data show heavy contaminations of milk samples with TBC indicator (73.6%), while SCC in only 6.4% samples were over the recommended levels by the Iranian standard. Therefore, it is necessary to take attention in order to control of these microbial parameters especially TBC during of milk production to avoid the potential risk of high microbial contamination.

**Specifications table**

**Table:** 

Subject area	*Microbiology*
More specific subject area	*Raw milk and microbial quality.*
Type of data	*Table, figure*
How data was acquired	*BactoScan (FOSS, Denmark), and Fossomatic (FOSS, Denmark) equipment*
Data format	*Raw, analyzed.*
Experimental factors	*Raw milk samples were taken from collection centers and stored within the sterile bottles at 4 °C and then transported to the laboratory. In the laboratory, the samples were maintained below 4 °C until analysis for microbial parameters*[1].
Experimental features	*Total Bacterial Count (TBC) and Somatic Cell Count (SCC) were determined using BactoScan and Fossomatic equipment respectively.*
Data source location	*East Azerbaijan province, Iran*
Data accessibility	*Data are available in this article*
Related research article	I. Stulova, S. Adamberg, T. Krisciunaite, M. Kampura, L. Blank, T.-M. Laht, Microbiological quality of raw milk produced in Estonia., Lett. Appl. Microbiol. 51 (2010) 683–90. doi:10.1111/j.1472-765X.2010.02951.x[3].

**Value of the data**

• TBC and SCC are the important factors to evaluation of the microbiological quality of raw milk, therefore, these data can be used for assessment of milk quality.
• Data from this research can be used for determination of the microbial quality of raw milk by the Food and Drug Administration, Iran.
• Data shown here can be useful for microbial evaluation of raw milk by the Ministry of Agriculture, Iran.

## 1 Data

The data available in Table 1, Table 3 show the total bacterial count (TBC) and somatic cell count (SCC) indicators of raw cow milk from 30 collection centers during 12 months respectively. In addition, the status of measured parameters is shown in Table 2, Table 4. Samples were measured during a month long period and averages were reported separately for each collection center.

**Table 1:** Mean values of TBC (Log CFU/mL) in raw milk samples.

**Collection center**	**Jan**	**Feb**	**Mar**	**Apr**	**May**	**Jun**	**Jul**	**Aug**	**Sep**	**Oct**	**Nov**	**Dec**
**1**	6.66	6.71	6.15	6.48	6.86	7.07	6.50	6.74	6.53	6.40	6.33	6.51
**2**	6.15	6.08	5.82	5.62	5.89	6.01	6.59	5.64	5.81	6.34	6.17	6.28
**3**	5.89	5.91	5.47	5.60	6.21	6.38	6.72	5.83	5.96	5.51	5.41	5.48
**4**	5.55	5.45	5.06	5.51	5.75	6.34	5.68	5.25	5.01	5.56	5.55	5.55
**5**	5.65	5.91	5.43	5.30	5.54	5.77	5.96	5.92	6.65	5.89	5.56	5.60
**6**	7.34	7.60	7.61	7.67	7.68	7.67	7.78	7.70	7.62	7.65	7.36	7.40
**7**	6.97	7.26	6.98	6.92	7.10	7.40	6.94	7.11	6.84	7.22	6.95	6.93
**8**	6.76	7.00	6.75	6.80	7.03	7.00	6.67	6.85	6.68	6.85	6.60	6.54
**9**	6.23	6.69	7.53	7.35	7.13	6.52	6.22	6.39	6.04	6.60	6.09	6.05
**10**	5.59	6.42	5.35	5.37	5.41	5.49	5.93	5.68	6.18	6.02	5.70	5.81
**11**	6.70	6.58	6.62	5.15	5.69	6.39	5.66	6.39	5.41	5.75	7.13	6.78
**12**	7.05	7.59	7.27	7.36	7.40	7.49	7.59	7.58	7.48	7.50	7.58	7.65
**13**	5.64	6.38	5.00	5.15	5.33	5.53	5.50	5.58	4.97	6.00	6.17	5.76
**14**	7.24	7.57	7.36	7.20	7.26	7.34	7.38	7.33	7.25	7.34	7.16	7.29
**15**	6.09	7.52	7.43	7.01	7.24	7.44	7.58	7.41	7.26	7.38	7.27	7.28
**16**	7.23	6.91	6.52	5.50	6.89	6.86	6.07	7.17	6.09	5.96	6.21	6.91
**17**	6.88	6.29	6.02	6.10	6.00	5.50	6.12	6.23	5.62	6.27	6.04	6.58
**18**	6.99	7.44	7.23	7.24	7.48	7.57	7.54	7.44	7.14	7.26	6.72	6.79
**19**	7.23	7.46	7.41	7.40	7.49	7.51	7.46	7.48	7.36	7.52	7.10	7.24
**20**	6.89	6.96	6.91	6.80	6.45	6.91	6.99	7.08	6.91	6.80	6.48	6.81
**21**	6.11	7.08	5.27	5.17	5.67	5.90	6.75	6.76	6.07	6.14	6.07	5.82
**22**	6.54	6.00	5.82	5.44	5.93	5.45	5.80	5.51	5.48	5.83	6.37	6.17
**23**	6.20	6.74	6.08	5.70	6.79	5.91	5.90	5.60	5.57	6.08	5.63	6.02
**24**	6.71	7.85	7.14	7.11	6.95	7.29	7.11	6.73	6.60	7.05	7.07	6.66
**25**	7.64	7.75	7.62	7.58	7.62	7.84	7.67	7.84	7.72	7.66	7.53	7.43
**26**	6.96	7.24	6.81	6.81	7.18	7.04	7.34	7.22	7.14	7.33	7.00	7.05
**27**	8.50	7.61	7.51	7.62	7.74	7.86	7.90	7.88	7.78	7.64	7.37	7.41
**28**	6.25	6.18	5.75	5.64	5.75	5.51	5.84	5.74	5.51	6.57	5.81	6.05
**29**	7.09	6.82	6.72	6.71	6.25	6.68	6.32	5.98	5.77	7.13	7.34	7.03
**30**	6.84	7.26	6.99	7.16	7.32	7.38	7.58	7.40	7.18	7.18	6.73	6.60

**Table 2:** Status of TBC in raw milk samples.

Milk ranking	Range (Log CFU/mL)	%
Excellent	≤4.48	–
First-grade	4.48–5	0.6
Second-grade	5–5.70	15.8
Third-grade	5.70–6	10.0
Non-standard	>6	73.6

**Table 3:** Mean values of SCC (Log Cell/mL) in raw milk samples.

**Collection center**	**Jan**	**Feb**	**Mar**	**Apr**	**May**	**Jun**	**Jul**	**Aug**	**Sep**	**Oct**	**Nov**	**Dec**
**1**	5.52	5.48	5.62	5.71	5.49	5.63	5.63	5.72	5.51	5.59	5.42	5.47
**2**	5.38	5.60	5.15	5.21	5.35	5.27	5.29	5.71	5.39	5.41	5.43	5.32
**3**	5.22	5.32	5.11	4.89	5.34	5.09	5.26	4.98	5.37	5.53	5.54	5.20
**4**	5.37	5.22	5.11	5.46	5.32	5.31	5.26	5.11	5.03	5.06	5.09	5.24
**5**	5.58	5.78	5.63	5.63	5.54	5.66	5.56	5.64	5.75	5.72	5.57	5.87
**6**	5.17	5.25	5.38	5.28	5.17	5.34	5.43	5.78	5.35	5.58	5.33	5.30
**7**	5.59	5.66	5.60	5.65	5.63	5.69	5.64	5.54	5.42	5.66	5.47	5.65
**8**	5.48	5.51	5.43	5.52	5.45	5.53	5.43	5.40	5.36	5.43	5.42	5.43
**9**	5.50	5.65	5.02	5.40	5.24	5.08	5.08	5.25	5.49	5.69	5.71	5.87
**10**	5.55	5.82	5.33	5.39	5.27	5.29	5.25	5.07	5.24	5.44	5.52	5.64
**11**	5.47	5.42	5.28	5.37	5.19	5.17	5.21	5.20	5.02	5.31	5.25	5.42
**12**	5.79	5.66	5.42	5.42	5.35	5.51	5.53	5.56	5.57	5.60	5.38	5.60
**13**	5.46	5.61	5.47	5.41	5.29	5.39	5.44	5.35	5.29	5.32	5.28	5.38
**14**	5.41	5.44	5.18	5.34	5.29	5.40	5.19	5.13	5.11	5.50	5.30	5.34
**15**	6.59	5.61	5.44	5.59	5.47	5.57	5.48	5.50	5.47	5.67	5.55	5.60
**16**	5.59	5.56	5.41	5.53	5.36	5.30	5.31	5.42	5.31	5.30	5.47	5.60
**17**	5.50	5.50	5.40	5.35	5.34	5.28	5.26	5.24	5.46	5.50	5.61	5.62
**18**	5.39	5.35	5.52	5.54	5.46	5.40	5.45	5.44	5.40	5.57	5.33	5.36
**19**	5.36	5.46	5.41	5.43	5.44	5.48	5.55	5.59	5.52	5.69	5.45	5.46
**20**	5.85	5.77	5.67	5.78	5.44	5.62	5.30	5.40	5.44	5.41	5.15	5.61
**21**	5.24	5.43	5.34	5.42	5.30	5.40	5.48	5.34	5.25	5.20	4.93	5.17
**22**	5.71	5.84	5.41	5.55	5.40	5.37	5.42	5.27	5.42	5.38	5.50	5.50
**23**	5.41	5.47	4.79	5.21	5.11	5.23	5.17	5.27	5.34	5.53	5.55	5.66
**24**	5.64	5.43	5.72	5.76	5.63	5.64	5.79	5.44	5.74	5.64	5.51	5.54
**25**	5.25	5.29	5.26	5.46	5.33	5.34	5.33	5.27	5.41	5.62	5.15	5.12
**26**	5.31	5.23	5.24	5.31	5.24	5.44	5.52	5.31	5.54	5.60	5.55	5.25
**27**	5.60	5.78	5.34	5.32	5.43	5.60	5.46	5.43	5.41	5.51	5.56	5.48
**28**	5.09	5.38	5.33	5.48	5.20	5.31	5.33	5.50	5.65	5.61	5.46	5.24
**29**	5.26	5.46	5.12	5.22	5.36	5.21	5.41	5.36	5.50	5.54	5.28	5.27
**30**	5.56	5.35	4.75	5.07	5.15	5.27	5.17	5.17	5.30	5.11	5.39	5.48

**Table 4:** Status of SCC in raw milk samples.

Milk ranking	Range (Log cell/mL)	%
Excellent	≤5	1.4
First-grade	5–5.30	25.6
Second-grade	5.30–5.60	56.9
Third-grade	5.60–5.70	9.7
Non-standard	>5.70	6.4

## 2 Experimental design, materials, and methods

### 2.1 Study area description

**Fig. 1:**
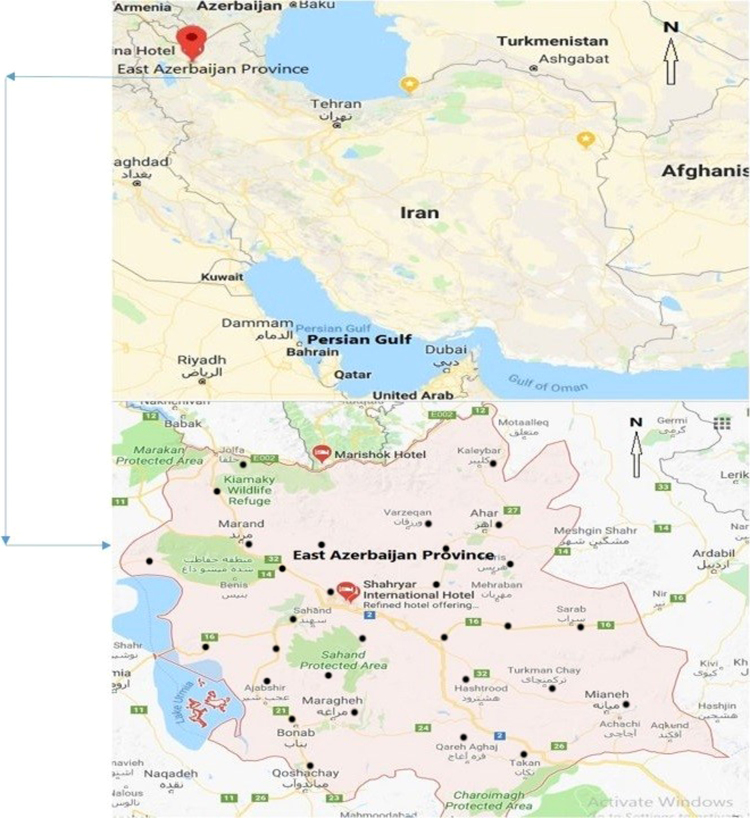
Location of the study area.

The center of East Azerbaijan province is the Tabriz that located at 46°18′ 13.47" N and 38°4′ 42.52" E and is 1401 m above sea level. The province located in the North West of Iran Fig. 1. According to the census of Iran in 2017 the population of this province was 3,900,000 people.

### 2.2 Determination of microbiological contaminant in raw milk

Here, samples were collected from 30 collection centers selected in different regions of East Azerbaijan province, Iran. A total of 10,800 samples (each sample 250 ml) were taken every morning during a year from January–December 2017 to test for microbial quality. The samples were transported to the laboratory in sterile bottles at 4 °C. Then, we assessed the microbial indicators within 4 h of collection.

In the laboratory, the analyses of the milk microbial composition included TBC and SCC and the samples were divided into two vials. Then, half of the vials were used for TBC analysis by BactoScan and the other half were analyzed for SCC using the Fossomatic. According to the Institute of Standards and Industrial Research of Iran, the standard limits for TBC in raw milk were divided into four groups (excellent ≤ 4.48, First-grade 4.48–5, Second-grade 5–5.70, and Third-grade 5.70–6 log_10_ CFU/ml), and four SCC groups (excellent ≤ 5, First-grade 5–5.30, Second-grade 5.30–5.60, and Third-grade 5.60–5.70 log_10_ cell/ml) [1], [2], [3], [4], [5], [6], [7], [8], [9], [10], [11]. Statistical analyses were carried out using SPSS software, version 22. The results of TBC and SCC were expressed as CFU/ml and Cell/ml respectively, in addition, transformed into base-10 logarithm.

## References

[1] Cassoli L.D., Lima W.J.F., Esguerra J.C., Da Silva J., Machado P.F., Mourão G.B. (2016). Do different standard plate counting (IDF/ISSO or AOAC) methods interfere in the conversion of individual bacteria counts to colony forming units in raw milk?. J. Appl. Microbiol..

[2] Numthuam S., Hongpathong J., Charoensook R., Rungchang S. (2016). Method development for the analysis of total bacterial count in raw milk using near-infrared spectroscopy. J. Food Saf..

[3] Stulova I., Adamberg S., Krisciunaite T., Kampura M., Blank L., Laht T.-M. (2010). Microbiological quality of raw milk produced in Estonia. Lett. Appl. Microbiol..

[4] LeJeune J.T., Rajala‐Schultz P.J. (2009). Unpasteurized milk: a continued public health threat. Clin. Infect. Dis..

[5] Hill B., Smythe B., Lindsay D., Shepherd J. (2012). Microbiology of raw milk in New Zealand. Int. J. Food Microbiol.

[6] Marcondes M.I., Jácome D.C., Lopes A., Rennó N., Clarissa A. (2014). Evaluation of raw milk quality in different production systems and periods of the year Milk is one of the most complete foods in nutritional. Rev. Bras. Zootec..

[7] Espeche M.C., Pellegrino M., Frola I., Larriestra A., Bogni C., Nader-macías M.E.F. (2012). Anaerobe lactic acid bacteria from raw milk as potentially beneficial strains to prevent bovine mastitis. Anaerobe..

[8] Li N., Wang Y., You C., Ren J., Chen W., Zheng H., Liu Z. (2018). Variation in raw milk microbiota throughout 12 months and the impact of weather conditions. Sci. Rep..

[9] Reguillo L., Hernández M., Barrientos E., Perez-Rodriguez F., Valero A. (2018). Evaluation of the influence of frequency of milk collection and milking dayshift on the microbiological quality of raw milk. J. Food Qual..

[10] Arias R., Oliete B., Ramón M., Arias C., Gallego R., Montoro V., Gonzalo C., Pérez-Guzmán M.D. (2012). Long-term study of environmental effects on test-day somatic cell count and milk yield in Manchega sheep. Small Rumin. Res..

[bib11] 11*Industrial Research and Standard Institute of Iran, ISIRI NO: 2406, 1st Edition.*

